# How a Needle Travels

**Published:** 1885-02

**Authors:** 


### HOW A NEEDLE TRAVELS.

### TEN YEARS AFTER TREADING ON A NEEDLE IT IS EXTRACTED.

The fact that foreign substances travel through the human body having been doubted, the following well authenticated case will be of considerable interest to the medical profession, clearly proving beyond a doubt that foreign substances not only travel through the human system, but also that the acid of the blood has no perceptible effect on steel which is internally subject to its action for a number of years: In July, 1874, Mrs. McDonald, one of the oldest residents of Harlem, and a lady owning considerable real estate in the city, accidentally trod upon a needle, which entered the foot at the base of the "phlanges" of the great toe, causing intense inflammation. Two well-known doctors were called in, and after two operations they were unable to find the needle. For four or five months Mrs. McDonald was unable to use her foot, till frightened by a call of fire she left her bed, and two days afterwards felt intense pain in the lower part of the calf of the leg. For over two years she suffered from a pricking sensation up the leg, when, two and a half years after the needle had entered the foot, it ceased troubling her and was forgotten.

About the middle of last month a sharp stinging pain was felt at the point of the ensiform cartilage, and after intense suffering a doctor was called in, who discovered that a foreign substance existed about one and a half inches above and one inch to the right of the umbilicus. An operati on was decided on and the delicate and dangerous operation successfully performed. The needle was extracted with a pair of dressing forceps and was found to be a No. 6 cambric needle in perfect condition, of a dark blue-black color, the point being still sharp, and the eye, although filled with hard matter, still perfect. The needle is in possession of Dr. M. W. Brooks, who attended the case and performed the operation. Mrs. McDonald, who is nearly 60 years of age, is out of danger and is expected to be about in a few days.—*Ex.*

